# Nutrient Calculation Accuracy, Expert-Rated Clinical Appropriateness and Safety Indicators, and Reproducibility of Large Language Model-Generated Diet Plans for Polyendocrine Metabolic Ovarian Syndrome: A Real-Case-Based Evaluation

**DOI:** 10.3390/nu18193205

**Published:** 2026-09-28

**Authors:** Ayşenur Çalık, Pınar Ece Demiray, Ayşe Betül Bilen, Gülen Ecem Kalkan, Muazzez Garipağaoğlu

**Affiliations:** 1 Department of Nutrition and Dietetics, Faculty of Health Sciences, Istanbul Atlas University, Istanbul 34403, Türkiye; pinarece.karakas@atlas.edu.tr (P.E.D.); ayse.demirbas@atlas.edu.tr (A.B.B.); gulen.kalkan@atlas.edu.tr (G.E.K.); 2 Department of Nutrition and Dietetics, Faculty of Health Sciences, Fenerbahçe University, Istanbul 34758, Türkiye; muazzez.garipagaoglu@fbu.edu.tr

**Keywords:** large language models, artificial intelligence, personalized nutrition, polyendocrine metabolic ovarian syndrome, polycystic ovary syndrome, diet planning, reproducibility, clinical appropriateness

## Abstract

**Background/Objectives:** This study evaluated the nutrient calculation accuracy, reproducibility, and expert-rated clinical appropriateness and safety indicators of three-day diet plans generated for women with polycystic ovary syndrome (PCOS), recently proposed to be termed polyendocrine metabolic ovarian syndrome (PMOS). **Methods:** Anonymized data from six women with heterogeneous PMOS profiles, purposively selected from a single clinic, were converted into standardized Turkish prompts and submitted to ChatGPT-4, Claude Sonnet 4.6, Gemini 2.5 Flash, and Mistral 2.0 through free web interfaces. Three independent generations per case–model combination yielded 216 daily menus. LLM-reported nutrient values were compared with BeBiS 9.0 calculations. Reproducibility was assessed using intraclass correlation coefficients (ICCs). Two experts independently evaluated the first generated plan for each case–model combination using a study-specific clinical appropriateness rubric and red-flag checklist. **Results:** No model achieved consistently low error across nutrients. Claude Sonnet 4.6 showed the lowest percentage errors for energy, carbohydrate, and fiber; ChatGPT-4 for protein; and Mistral 2.0 for fat. Gemini 2.5 Flash showed the largest percentage errors across all nutrients and a mean energy bias of +531.1 kcal. ChatGPT-4 showed comparatively higher plan-level reproducibility, while the remaining models generally showed poor reproducibility. Claude Sonnet 4.6 had the highest descriptive clinical appropriateness scores and Gemini 2.5 Flash the lowest, but no pairwise differences remained significant after Holm correction. Gemini 2.5 Flash generated the most confirmed study-specific red flags (*n* = 28), with all six cases meeting the criterion for the “masked low-energy” indicator. **Conclusions:** LLM-generated diet plans showed model-dependent calculation errors and limited reproducibility, and the present findings do not support their independent or unsupervised use as individualized clinical nutrition recommendations.

## 1. Introduction

Polycystic ovary syndrome (PCOS) is one of the most common endocrine disorders affecting women of reproductive age [1]. It is estimated to affect approximately 170 million women worldwide and is recognized as a complex, multisystem metabolic condition associated not only with menstrual irregularities, hyperandrogenism, and ovulatory dysfunction, but also with insulin resistance, dyslipidemia, abdominal obesity, impaired glucose metabolism, and increased cardiometabolic risk [1,2,3]. In line with this broader understanding, a recent international consensus published in *The Lancet* proposed renaming the condition as polyendocrine metabolic ovarian syndrome (PMOS), emphasizing that the disorder is not limited to ovarian dysfunction but also encompasses multiple endocrine and metabolic abnormalities [3]. Therefore, in accordance with the updated terminology, the term PMOS will be used throughout the remainder of this manuscript.

Medical nutrition therapy is regarded as a core component of PMOS management, with an important role in addressing both metabolic and clinical manifestations. Current guidelines emphasize energy balance, reduced intake of simple carbohydrates, increased dietary fiber, healthier dietary fat patterns, and the development of sustainable eating habits [1,4]. However, given the heterogeneous nature of PMOS, nutritional needs may differ substantially across individuals. Therefore, dietary management should not rely on a uniform approach but should be individualized according to the patient’s metabolic profile, while remaining balanced, feasible, and clinically safe [5].

In recent years, large language models (LLMs) have been increasingly used for health-related information generation and decision-support processes. LLM-based chatbots, in particular, are widely used by individuals to obtain health information, interpret symptoms, and seek nutrition-related advice [6,7]. Their ability to generate personalized diet plans within a short time offers considerable potential for digital health applications, especially in the management of non-communicable chronic diseases [6].

Although the existing literature suggests that LLM-based systems may have potential applications in dietary planning, several studies have reported inaccuracies in energy and nutrient calculations, as well as deviations from clinical guidelines [8,9,10]. Such limitations may be particularly important in PMOS, a metabolically sensitive condition in which errors in energy or macronutrient estimation, or recommendations that fail to consider the individual metabolic risk profile, may have clinically relevant implications [5].

Current research on artificial intelligence (AI) in PMOS/PCOS has primarily focused on diagnostic support, risk prediction, classification, image analysis, and patient education [11,12]. Within the nutrition field, existing studies have mostly addressed AI-based recommendation systems, requirements for mobile health applications, food image recognition approaches, and quality assessments of chatbot-generated responses [13,14]. However, evidence remains limited regarding LLM-generated personalized diet plans for individuals with PMOS, particularly studies based on real clinical case data that jointly evaluate nutrient calculation accuracy, clinical appropriateness, safety indicators, and reproducibility.

To our knowledge, this is the first study to jointly evaluate the nutrient calculation accuracy, reproducibility, and expert-rated clinical appropriateness and safety indicators of diet plans generated by multiple LLMs using real clinical case data from individuals with PMOS.

This study aimed to evaluate the nutrient calculation accuracy, clinical appropriateness, metabolic risk alignment, safety indicators, and reproducibility of three-day diet plans generated by four different LLMs using real clinical case data. In this way, this study seeks to characterize the reliability and limitations of LLM-generated nutrition recommendations and to identify potential concerns relevant to their future evaluation in clinical nutrition and the dietary management of PMOS.

## 2. Materials and Methods

### 2.1. Study Design

This single-center, retrospective, real-case-based methodological study evaluated diet plans generated by LLMs for individuals with PMOS. Anonymized clinical and laboratory data from six women diagnosed with PMOS and followed in a private clinic were used. Given the heterogeneous clinical and metabolic nature of PMOS, cases were purposively selected to represent different metabolic risk profiles rather than a uniform patient group. The six cases were selected by the research team from a larger pool of clinic patients with PMOS to jointly span variation in adiposity (body mass index (BMI)), insulin resistance (HOMA-IR), thyroid function, and clinical manifestations (e.g., hirsutism, oligomenorrhea), reflecting the principal axes of metabolic and clinical heterogeneity in PMOS. Given the exploratory, methodological aim of the study, this selection was not based on a systematic or randomized screening of the full clinic population. These data were converted into standardized case prompts and submitted to different LLMs to generate three-day diet plans for each case.

The generated diet plans were not implemented in patients, were not used for clinical decision-making, and were analyzed solely for research purposes. The study assessed the nutrient calculation accuracy, clinical appropriateness, and within-model reproducibility of the responses generated from a single user prompt.

### 2.2. Case Data and Prompt Development

The case prompts were developed to include age, height, body weight, BMI, physical activity level, clinical symptoms, waist circumference, and laboratory parameters. Physical activity level was assessed using the International Physical Activity Questionnaire (IPAQ) as part of the original clinical assessment. Participants were classified according to the IPAQ scoring criteria, and the recorded physical activity category was subsequently incorporated into the standardized case prompts. To reflect the clinical and metabolic heterogeneity of PMOS, the cases included varying profiles in terms of adiposity, insulin resistance, lipid parameters, thyroid function, and clinical manifestations. An overview of the clinical and metabolic characteristics of the six cases is presented in Figure 1. The complete clinical and laboratory characteristics of each case are presented in Supplementary Table S1. The information provided to the LLMs was restricted to the clinical, anthropometric, and laboratory variables available in the standardized study prompts. Medication and supplement use, food allergies or intolerances, habitual dietary intake, detailed food preferences, pregnancy status, specific weight-management goals, and ongoing therapeutic interventions were not systematically incorporated into the prompts; consequently, the generated plans should not be interpreted as equivalent to recommendations derived from a comprehensive individualized medical nutrition therapy assessment.

For each case, every model was asked to generate a three-day diet plan using foods commonly consumed and easily accessible in Türkiye. The models were also asked to specify the amount of each food item in grams and to calculate the total daily energy, protein, fat, carbohydrate, and fiber content for each day.

### 2.3. LLM Selection and Generation Protocol

Four different large language models were evaluated in this study: ChatGPT-4 [15], Claude Sonnet 4.6 [16], Gemini 2.5 Flash [17], and Mistral 2.0 [18]. The models were reported according to the labels displayed in their respective web interfaces at the time of data collection. Data generation was conducted between 24 and 28 March 2026 in Türkiye. All models were accessed through their official publicly available free web interfaces. No paid subscription, API access, or developer settings were used. This approach was intended to evaluate the performance of the models under standard access conditions that reflect how they are commonly used by real-world users. Model labels, access dates, and interface information were recorded during data collection.

To minimize the potential influence of previous user interactions, chat history, and personalization effects, new and independent user accounts were created for each generation process. Prompts were submitted to the models in Turkish, reflecting the language in which the clinical data were originally recorded and in which the models are commonly used by Turkish-speaking users; English translations of the prompts are provided in Supplementary Table S2 for reporting purposes. Before data collection, no prior interactions related to health, nutrition, or diet were conducted in these accounts. Each case–model–repeat combination was performed in an independent session, without transferring any context from previous conversations, and each generation was initiated in a clean chat window.

Regarding generation parameters, the free web interfaces did not allow user-adjustable settings such as temperature, top-p, seed, or other sampling parameters. Therefore, all models were used under their default web-interface settings, and no manual temperature adjustment was applied during the generation process.

Three independent repetitions were performed for each model–case combination. Each repetition was conducted in a separate session, and no additional clarification, correction, guidance, or follow-up prompt was provided after the initial response. All outputs were recorded verbatim, and no corrections or modifications were made by the researchers.

### 2.4. Output Dataset

For each case, three-day diet plans were obtained from four models across three independent repetitions. Accordingly, a total of 216 daily menus were generated, corresponding to 6 cases × 4 models × 3 repetitions × 3 days. All generated menus were included in the nutrient calculation accuracy and within-model reproducibility analyses. Clinical appropriateness and study-specific safety indicators were evaluated using the first repetition of each case–model combination, as described below.

### 2.5. Nutrient Analysis

All three-day diet plans generated by the LLMs were analyzed using BeBiS 9.0 (Nutrition Information System) [19], a computerized dietary analysis software used to calculate the energy and nutrient composition of foods and dietary records. In the present study, BeBiS 9.0 was used as an independent nutrient-composition calculation tool to determine the energy, protein, fat, carbohydrate, and fiber content of the generated menus; it was not used to determine patient-specific nutritional requirements. Each menu day was assessed separately, and total daily energy, protein, fat, carbohydrate, and fiber values were calculated. BeBiS coding was performed by a single dietitian researcher who was different from the two dietitian researchers responsible for submitting the standardized prompts to the LLMs and was blinded to the identity of the model that generated each menu. The food amounts and gram values specified in the model-generated menus were used as the basis for the analyses.

When entering foods into BeBiS 9.0, the gram amounts provided in the model outputs were used directly. The daily energy and nutrient values reported by the LLMs were then compared with the corresponding values independently calculated using BeBiS 9.0. When a generated food or mixed dish could correspond to more than one BeBiS entry, the entry most closely matching the food description and composition provided in the generated menu was selected. Because the standardized prompts explicitly specified a Turkish dietary context, Turkish mixed dishes and household foods were coded according to the corresponding standard recipes and ingredient compositions available in BeBiS. This comparison was used to assess the quantitative nutrient calculation accuracy of the models, defined as the agreement between LLM-declared nutrient values and the independently recalculated nutrient composition of the same menus, rather than the nutritional adequacy of the menus for individual patient requirements. For energy, protein, fat, carbohydrate, and fiber, the absolute difference, percentage error, and mean deviation between the model-reported values and BeBiS-derived values were calculated.

### 2.6. Expert-Rated Clinical Appropriateness and Safety Indicators

Clinical appropriateness and potential safety concerns of the generated dietary plans were assessed using a 10-item expert rubric developed by the research team, covering energy plausibility and avoidance of excessively low energy provision, carbohydrate and fat quality, fiber/protein adequacy, alignment with the patient’s metabolic-laboratory profile, absence of supplement/medication dosing advice, absence of unsubstantiated or dogmatic claims, clarity of reported gram and macronutrient values, internal consistency of reported values, and overall comprehensibility with appropriate clinical referral. Each item was scored on a 0–2 scale (0 = absent/incorrect, 1 = partial/insufficient, 2 = present/correct), yielding a raw total score ranging from 0 to 20. The raw total score was standardized to a 0–100 scale using the formula: (raw score/20) × 100. In parallel, an 8-item study-specific red-flag checklist was applied independently of the total score to identify plan characteristics warranting different levels or types of professional concern. For interpretation, the predefined indicators were grouped into four categories: (1) quantitative discrepancies of particular concern, comprising excessively low energy content and the “masked low-energy” indicator; (2) guideline deviations requiring professional review, comprising unjustified very-low-carbohydrate content (<130 g/day) and imbalanced total fat distribution (>40% or <20% of total energy); (3) potentially unsafe recommendations or clinically concerning content, comprising supplement/medication dosing recommendations, treatment/cure claims, and recommendations conflicting with the patient’s metabolic profile; and (4) an evaluability/validation concern, defined as unclear gram quantities precluding software-based validation. These categories were introduced to distinguish the nature and potential severity of the indicators and were not intended to imply that all checklist items represented intrinsically unsafe recommendations.

The 10-item expert rubric and 8-item red-flag checklist were developed prospectively by the research team during the study-planning phase, before evaluation of the LLM-generated outputs. Item selection was informed by clinically relevant nutritional and safety considerations and by established clinical nutrition reference values. To improve content relevance and clarity, the proposed items and evaluation criteria were reviewed by two dietitian faculty members who were not otherwise involved in the study, and their feedback was incorporated before the expert-rating process. However, the rubric and red-flag checklist were study-specific instruments and did not undergo formal psychometric validation or formal pilot validation. Accordingly, they were used as structured, expert-rated indicators of clinical appropriateness and potential safety concerns rather than as validated measures of clinical safety. A BeBiS-calculated energy intake of <1200 kcal/day was classified as low energy because diets providing 800–1200 kcal/day are recognized as low-energy diets that should be implemented within structured, professionally supervised management [20]. This threshold was used as a study-specific screening criterion for potentially concerning low energy provision and was not intended to represent an individualized estimate of energy inadequacy or a patient-specific energy requirement. Unjustified carbohydrate intake of <130 g/day was flagged with reference to the adult recommended dietary allowance (RDA) for carbohydrate [21] and was classified as a guideline deviation requiring professional review rather than as an intrinsically unsafe intake level. For total fat, <20% or >40% of energy was used as a deliberately broad, study-specific screening range. The lower boundary was informed by the adult acceptable macronutrient distribution range of 20–35% of energy, whereas the upper boundary was extended to 40% to avoid flagging modest deviations [22]. Values outside this study-specific range were classified as guideline deviations requiring professional review rather than as intrinsically unsafe dietary recommendations, because their clinical relevance depends on factors such as fat quality, total energy intake, and individual clinical context. A study-specific “masked low-energy” indicator was operationally defined as a plan reporting ≥1400 kcal/day while providing <1200 kcal/day according to BeBiS analysis, thereby identifying potentially misleading discrepancies between apparently adequate declared energy and independently verified low energy content.

Rubric and red-flag evaluations were performed by two dietitians with expertise in clinical nutrition, who were independent of the study authors and blinded to the identity of the model that generated each plan. Rubric evaluation was conducted on the first repetition of each model–case combination (*n* = 24). All 24 plans were independently evaluated by two expert raters, resulting in 48 expert ratings. A red flag was considered confirmed only when both raters independently identified it for the same plan. This conservative operational definition was used to distinguish red flags independently identified by both raters from concerns identified by only one rater. To assess the sensitivity of the findings to this definition and to make rater disagreements transparent, we additionally performed a descriptive analysis in which a potential red flag was counted when it was identified by at least one of the two raters. Detailed item-level rubric scores and red flag coding are provided in Supplementary Tables S3 and S4, respectively, together with the corresponding item definitions and coding criteria in the table footnotes. Item-level inter-rater agreement results for the 10-item rubric and the red-flag checklist are presented in Supplementary Tables S5 and S6, respectively.

### 2.7. Statistical Analysis

Statistical analyses were performed using SPSS version 25.0 [23] and Jamovi Software version 2.7 [24], as appropriate. Continuous variables were expressed as mean ± standard deviation. For inferential comparisons between LLM-declared and BeBiS-calculated nutrient values, the clinical case was treated as the independent analytical unit to account for the non-independence of menu days generated within the same plan. In the primary analysis, which was based on the first generated response for each case–LLM combination, nutrient values were averaged across the three menu days within each case–model combination, yielding six independent case-level observations per model. The normality of the case-level differences between LLM-declared and BeBiS-calculated values was assessed using the Shapiro–Wilk test. Paired-samples *t*-tests were used when the difference variable was normally distributed; otherwise, Wilcoxon signed-rank tests were applied.

A sensitivity analysis incorporating all three repeated generations was also conducted. For this analysis, nutrient values were averaged across the three generations and three menu days within each case–model combination (nine daily menus per case–model combination), again yielding six independent case-level observations per model. This analysis was used to determine whether the direction and pattern of the primary findings were robust to inclusion of all repeated generations. Daily-menu-level observations were retained for descriptive error metrics. Reproducibility was evaluated separately at the three-day plan level, as described below.

The reproducibility of LLM-generated diet plans across the three independent generations was assessed at the three-day plan level. For each case–model–generation combination, BeBiS-calculated energy, carbohydrate, protein, fat, and fiber values were averaged across the three days of the generated plan. Agreement among the three independent generations was then assessed across the six cases using intraclass correlation coefficients (ICC), calculated using a two-way mixed-effects model under an absolute-agreement definition; single-measure ICC values with 95% confidence intervals are reported. ICC values were interpreted according to the thresholds proposed by Koo and Li [25]: <0.50, poor; 0.50–0.75, moderate; 0.75–0.90, good; and >0.90, excellent reliability. This plan-level approach was used because corresponding individual menu days across independent generations were not assumed to represent identical dietary units. The magnitude of deviation between LLM-declared and BeBiS-calculated values was quantified using mean absolute percentage error (MAPE), mean absolute error (MAE), and root mean square error (RMSE), calculated as follows:

$$MAE=\frac{1}{n}\sum_{i=1}^{n}\left|AI_{i}-BeBiS_{i}\right|$$

$$MAPE=\frac{100}{n}\sum_{i=1}^{n}\left|\frac{AI_{i}-BeBiS_{i}}{BeBiS_{i}}\right|$$

$$RMSE=\sqrt{\frac{1}{n}\sum_{i=1}^{n}{\left(AI_{i}-BeBiS_{i}\right)}^{2}}$$

where $AI_{i}$ represents the nutrient value declared by the LLM, $BeBiS_{i}$ represents the corresponding independently calculated value, and n represents the number of observations included in the analysis.

Case-level cluster bootstrap 95% confidence intervals were obtained using 5000 resamples by sampling the six clinical cases with replacement while retaining all daily observations within each selected case. Agreement between LLM-declared and BeBiS-calculated energy values was assessed using a repeated-measures Bland–Altman approach accounting for within-and between-case variance components.

Inter-rater reliability for expert rubric scoring was assessed at both the item and total-score levels. Item-level agreement for the 10-item clinical appropriateness and safety-indicator rubric was evaluated using percentage agreement and quadratic weighted Cohen’s kappa. Item-level agreement for the binary red flag checklist was evaluated using percentage agreement and Cohen’s kappa. When kappa statistics could not be estimated because of limited variability or complete marginal imbalance, values were indicated with a dash. Agreement for the total clinical appropriateness and safety-indicator rubric score and total red flag checklist score was assessed using intraclass correlation coefficients (ICC), calculated using a two-way mixed-effects model under an absolute agreement definition; single-measure ICC values with 95% confidence intervals are reported. Overall differences in standardized clinical appropriateness scores and confirmed red-flag counts across the four models were evaluated using the Friedman test, with Kendall’s W reported as the effect-size measure. Significant omnibus tests were followed by pairwise Wilcoxon signed-rank tests with Holm correction. Differences in the proportion of cases containing at least one confirmed red flag were evaluated using Cochran’s Q test with exact permutation inference. Given the exploratory methodological design, the purposive six-case sample, and the absence of a prespecified single confirmatory hypothesis, all model–nutrient comparisons, nutrient calculation analyses, reproducibility analyses, and case-level analyses were considered exploratory. No multiplicity adjustment was therefore applied across these exploratory analyses; Holm correction was used only for the prespecified post hoc pairwise comparisons following significant omnibus tests. Accordingly, *p*-values from the exploratory analyses were interpreted descriptively and in conjunction with the magnitude and direction of observed differences, error metrics, and confidence intervals rather than as confirmatory evidence. A two-tailed *p*-value < 0.05 was used as a descriptive threshold for statistical significance.

### 2.8. Ethical Considerations

This study was approved by the Non-Interventional Scientific Research Ethics Committee of Istanbul Atlas University (meeting date: 23 March 2026; meeting no: 03; approval no: 47). The study had a retrospective, real-case-based, non-interventional design. Anonymized clinical and laboratory data from individuals diagnosed with PMOS and followed in a private clinic were used. No direct contact was made with patients, no new data were collected, and no dietary intervention was implemented. The diet plans generated by the large language models were not provided to patients, were not used in clinical decision-making, and were analyzed solely for research purposes. All data were evaluated anonymously, and no personally identifiable information was included in the research dataset.

## 3. Results

### 3.1. Comparison of AI-Declared and BeBiS-Calculated Values

Substantial discrepancies were observed between AI-declared and BeBiS-calculated energy and macronutrient values across the four models, although their magnitude and direction varied by model and nutrient. To account for the non-independence of the three daily menus within each generated plan, the primary inferential analysis was conducted at the case level by averaging the three menu days within each case–model combination, yielding six independent observations per model (Table 1).

At the case level, the magnitude and direction of the discrepancies varied across models and nutrients (Table 1). ChatGPT-4 showed its largest mean discrepancy for energy (−117.7 kcal), while the mean protein and fiber differences were small (−1.5 g and +0.1 g, respectively). Claude Sonnet 4.6 underestimated energy (−183.6 kcal), protein (−16.3 g), fat (−27.1 g), and fiber (−5.8 g), while overestimating carbohydrate (+31.1 g). Gemini 2.5 Flash showed the largest discrepancies, particularly for energy (+531.1 kcal) and carbohydrate (+86.7 g), with positive mean differences across all evaluated nutrients. Mistral 2.0 showed smaller mean discrepancies for energy (+60.3 kcal) and protein (+8.0 g), while overestimating carbohydrate (+32.2 g) and fiber (+5.6 g) and underestimating fat (−6.6 g). Corresponding paired-test *p*-values are provided in Table 1, but given the exploratory six-case design, interpretation focused primarily on the magnitude and direction of the case-level differences and the descriptive error metrics with confidence intervals reported in Section 3.2.

### 3.2. Accuracy Metrics

Table 2 summarizes the mean absolute error (MAE), mean absolute percentage error (MAPE), and root mean square error (RMSE) for each model and nutrient, together with case-level cluster bootstrap 95% confidence intervals. Among the evaluated cases and outputs, Claude Sonnet 4.6 and Mistral 2.0 most frequently showed the lowest errors according to the predefined accuracy metrics, whereas Gemini 2.5 Flash showed the largest errors across all evaluated nutrients.

The magnitude of the differences varied by nutrient. Gemini 2.5 Flash produced the largest carbohydrate errors, with a MAPE of 137.9% (95% CI: 117.2–166.3). Claude Sonnet 4.6 showed the lowest MAPE for energy (10.6%, 95% CI: 7.6–13.4), carbohydrate, and fiber among the evaluated models. ChatGPT-4 showed the lowest protein error (MAE = 13.2 g; MAPE = 13.8%), whereas Mistral 2.0 showed the lowest fat error (MAE = 11.6 g; MAPE = 16.0%).

The Bland–Altman [26] analysis of energy values, accounting for both within- and between-case variance components (Figure 2), supported the findings of the accuracy analysis. Gemini 2.5 Flash exhibited the largest positive bias (mean bias = +531.1 kcal), with limits of agreement almost entirely above zero (32.1 to 1030.2 kcal), indicating systematic overestimation of energy content across nearly all cases. Claude Sonnet 4.6 showed a moderate negative bias (−190.3 kcal) together with the narrowest limits of agreement (−492.2 to 111.6 kcal). ChatGPT-4 and Mistral 2.0 exhibited smaller bias magnitudes (−117.7 and +82.0 kcal, respectively) but wider limits of agreement, indicating greater variability in agreement across repeated menu generations. Across all models, the within-case standard deviation was comparable to or greater than the between-case standard deviation, suggesting that variability between repeated daily menus within the same clinical case contributed substantially to the overall disagreement.

### 3.3. Clinical Appropriateness and Study-Specific Safety Findings

Expert-rated clinical appropriateness and study-specific safety indicators evaluated using the first generated plan for each case–model combination, varied among the four LLMs (Table 3). Claude Sonnet 4.6 achieved the highest expert rubric scores, with a median standardized score of 92.5 (IQR: 90.0–96.9), followed by ChatGPT-4 [83.8 (80.6–86.9)]. Mistral 2.0 showed intermediate performance, whereas Gemini 2.5 Flash received the lowest rubric scores [62.5 (58.1–65.0)].

The overall model effect on expert rubric scores was statistically significant and large (Friedman χ^2^(3) = 15.61, *p* = 0.0014; Kendall’s W = 0.867). However, none of the pairwise Wilcoxon signed-rank comparisons remained statistically significant after Holm correction, likely reflecting the limited statistical power associated with the six matched cases.

Confirmed study-specific red flags were observed in all six ChatGPT-4 and Gemini 2.5 Flash plans, five of six Claude Sonnet 4.6 plans, and four of six Mistral 2.0 plans. Gemini 2.5 Flash generated the greatest number of confirmed red flags (*n* = 28), corresponding to a median of 5.0 (IQR: 4.25–5.0) per case. In contrast, Claude Sonnet 4.6 generated the fewest confirmed red flags (*n* = 7), with a median of 1.0 (IQR: 1.0–1.75) per case. ChatGPT-4 and Mistral 2.0 each generated nine confirmed red flags.

Confirmed red-flag counts differed significantly among models (Friedman χ^2^(3) = 12.21, *p* = 0.0067; Kendall’s W = 0.678). In contrast, the proportion of cases containing at least one confirmed red flag did not differ significantly (Cochran’s Q = 3.67, *df* = 3, exact permutation *p* = 0.626), reflecting the limited discriminatory value of this binary outcome because most case–model plans contained at least one confirmed red flag.

Inter-rater agreement for the expert evaluation was good at the total-score level. The single-measure ICC was 0.821 (95% CI: 0.633–0.918; *p* < 0.001) for the standardized rubric total score and 0.853 (95% CI: 0.680–0.935; *p* < 0.001) for the total red-flag checklist score. However, item-level agreement was heterogeneous. Agreement was low for Item 5 (metabolic profile alignment; 50% agreement, weighted κ = 0.090), Item 6 (absence of supplement/medication dosing; 83% agreement, weighted κ = 0.076), and Item 9 (internal consistency; 58% agreement, weighted κ = 0.104). These findings indicate that good reliability of the overall rubric score did not necessarily extend to all individual criteria. Detailed item-level agreement and kappa statistics are provided in Supplementary Tables S5 and S6.

### 3.4. Distribution of Confirmed Study-Specific Red Flags

The predefined red-flag indicators represented different types and levels of concern and should not be interpreted as equivalent clinical safety events; in particular, carbohydrate and total-fat threshold deviations were classified as guideline deviations requiring professional review. The distribution of confirmed study-specific red flags varied by both model and red-flag category (Figure 3). Gemini 2.5 Flash generated the largest number of confirmed study-specific red flags across the evaluated cases. All six Gemini-generated plans had BeBiS-calculated energy values below 1200 kcal and met the criterion for the study-specific “masked low-energy” indicator, in which the model-declared energy was at least 1400 kcal despite the independently calculated energy being below 1200 kcal. All Gemini-generated plans also met the study-specific criteria for guideline deviations requiring professional review, including unjustified carbohydrate intakes below 130 g/day and fat contributions outside the predefined range of 20–40% of total energy.

Unclear portion sizes were identified in three of the six Gemini 2.5 Flash plans and in one Mistral 2.0 plan. A definitive treatment- or cure-related claim was confirmed in one Gemini-generated plan. No confirmed medication or supplement dosing recommendation was identified for any model, and no recommendation was jointly rated as inconsistent with the corresponding metabolic profile.

Imbalanced fat distribution was the most frequently observed confirmed red flag across the evaluated models, occurring in all ChatGPT-4 and Gemini 2.5 Flash plans, five of six Claude Sonnet 4.6 plans, and four of six Mistral 2.0 plans. Unjustified carbohydrate intake below 130 g/day was also observed across all models but occurred most frequently in Gemini 2.5 Flash outputs.

As a descriptive sensitivity analysis, red-flag findings were also summarized using a more inclusive criterion in which identification by at least one rater was sufficient. Under the primary conservative definition requiring agreement by both raters, the total numbers of confirmed red flags were 9 for ChatGPT-4, 7 for Claude Sonnet 4.6, 28 for Gemini 2.5 Flash, and 9 for Mistral 2.0. When red flags identified by at least one rater were considered, the corresponding numbers of potential red flags increased to 11, 10, 36, and 15, respectively. Under this broader criterion, at least one potential red flag was identified in all six cases for each of the four LLMs, whereas the both-rater criterion identified at least one confirmed red flag in 6/6, 5/6, 6/6, and 4/6 cases, respectively. Thus, the broader definition increased the absolute number of identified concerns but did not alter the overall pattern of Gemini 2.5 Flash having the highest red-flag count. Detailed results are provided in Supplementary Table S7.

### 3.5. Reproducibility Across Repeated Prompts

Reproducibility across the three independent generations was evaluated at the three-day plan level by averaging the BeBiS-calculated nutrient values across the three days within each generation (Table 4 and Figure 4). Plan-level reproducibility varied substantially by model and nutrient.

Among the six evaluated cases, ChatGPT-4 demonstrated comparatively higher overall plan-level reproducibility than the other evaluated models. The point estimates fell within the “good” reliability range for protein (ICC = 0.825, 95% CI: 0.468–0.971) and energy (ICC = 0.756, 95% CI: 0.354–0.957), and within the moderate range for carbohydrate (ICC = 0.583, 95% CI: 0.119–0.915), fat (ICC = 0.582, 95% CI: 0.029–0.919), and fiber (ICC = 0.567, 95% CI: 0.016–0.915). However, the wide confidence intervals, attributable in part to the six-case sample, indicate substantial uncertainty around these reliability estimates.

In contrast, Claude Sonnet 4.6, Gemini 2.5 Flash, and Mistral 2.0 generally showed poor plan-level reproducibility. For Claude Sonnet 4.6, ICCs ranged from −0.128 for fat to 0.452 for energy; for Gemini 2.5 Flash, from −0.207 for fiber to 0.467 for energy; and for Mistral 2.0, from −0.078 for fat to 0.476 for protein. Most confidence intervals for these three models included zero, indicating substantial uncertainty in reproducibility estimates.

Overall, averaging nutrient composition across the complete three-day plans reduced the dependence of the reproducibility assessment on corresponding individual days across repeated generations. Under this plan-level approach, ChatGPT-4 showed comparatively greater reproducibility across all five evaluated nutrient parameters, whereas the remaining models generally demonstrated poor reproducibility.

Figure 4 visualizes the three-day plan-level reproducibility findings presented in Table 4. ChatGPT-4 showed comparatively higher ICC values across all five evaluated nutrient parameters, with the highest agreement observed for protein and energy. In contrast, Claude Sonnet 4.6, Gemini 2.5 Flash, and Mistral 2.0 showed predominantly poor plan-level reproducibility, with several ICC estimates close to or below zero.

### 3.6. Case-Level Error Patterns

Case-level MAPE values demonstrated substantial variation in nutrient calculation error across both clinical cases and LLMs (Figure 5). Gemini 2.5 Flash consistently exhibited the highest energy errors, with MAPE values ranging from 35% to 71% across all six cases. The same model also showed the largest carbohydrate and fiber errors, with carbohydrate MAPE ranging from 105% to 140% and fiber MAPE from 72% to 109%.

Protein errors showed a similar pattern, with Gemini 2.5 Flash producing the highest MAPE values in five of the six cases (21–42%). In contrast, fat errors varied more substantially across models. Gemini 2.5 Flash showed the largest fat errors in Cases 1 and 6, whereas Claude Sonnet 4.6 produced the largest fat errors in Cases 3 and 5. Mistral 2.0 generally exhibited lower fat errors than the other models in Cases 3, 4, and 6.

ChatGPT-4 generally exhibited lower energy and protein errors than the other models. However, its MAPE increased in Case 4, reaching 26% for energy and 56% for carbohydrate. Claude Sonnet 4.6 maintained relatively low energy errors across cases but showed larger carbohydrate and fat deviations, whereas Mistral 2.0 demonstrated intermediate error levels with comparatively higher carbohydrate and fiber errors in Cases 3, 4, and 6.

Overall, case-level error patterns differed substantially across nutrients and clinical cases. Gemini 2.5 Flash consistently exhibited the largest errors, particularly for energy, carbohydrate, and fiber, whereas no model maintained uniformly low error values across all six cases and evaluated nutrients.

### 3.7. Sensitivity Analysis Including All Repeated Generations

The sensitivity analysis incorporated all three repeated generations. To account for the nested structure of menu days and repeated generations, nutrient values were averaged across the three generations and three menu days within each case–model combination (nine daily menus per case–model combination), yielding six independent case-level observations per model for inferential comparisons (Supplementary Table S8).

The overall direction and model-specific pattern of the discrepancies remained broadly consistent with the primary case-level analysis. For ChatGPT-4, only fat differed significantly between LLM-declared and BeBiS-calculated values (*p* = 0.010), whereas energy, carbohydrate, protein, and fiber were not statistically significant (all *p* ≥ 0.066). Claude Sonnet 4.6 and Gemini 2.5 Flash showed significant differences across all evaluated parameters (all *p* ≤ 0.008). For Mistral 2.0, significant differences were observed for carbohydrate, protein, fat, and fiber (all *p* ≤ 0.048), whereas the difference in energy was not statistically significant (*p* = 0.233).

The accuracy metrics presented in Supplementary Table S9 were consistent with the primary findings. Gemini 2.5 Flash exhibited the largest percentage errors, particularly for carbohydrate and fiber, whereas ChatGPT-4 generally showed lower percentage errors. Claude Sonnet 4.6 and Mistral 2.0 demonstrated nutrient-specific error patterns. Overall, inclusion of all repeated generations did not materially change the direction or general model-specific pattern of the discrepancies.

## 4. Discussion

AI holds considerable potential in personalized nutrition because it can rapidly generate accessible dietary recommendations based on individuals’ clinical and metabolic characteristics. However, the findings of this study indicate that a dietary plan generated using individual-specific data may not always be nutritionally accurate, reproducible, or clinically reliable. In this study, three-day diet plans generated by four different LLMs were evaluated in terms of nutrient calculation accuracy, consistency across repeated generations, case-level error patterns, and expert-rated clinical appropriateness and study-specific safety indicators using real clinical data from individuals diagnosed with PMOS and a standardized access protocol reflecting real-world user conditions. Taken together, the findings showed that model performance varied across these dimensions and that none of the evaluated models produced sufficiently consistent and reliable outputs to support their independent and unsupervised use as personalized nutrition tools in this metabolically sensitive population. These findings provide methodological evidence regarding the current limitations of LLM-generated diet plans and inform their future evaluation in AI-driven personalized nutrition.

### 4.1. Nutrient Calculation Accuracy and Model-Level Differences

In the present study, model-specific discrepancies were identified between the energy and nutrient values reported by the LLMs and those independently calculated from the same dietary plans using BeBiS 9.0 [19]. Both the magnitude and direction of these discrepancies varied according to the model and nutrient examined. Importantly, no single model consistently achieved the highest accuracy across all nutrients. Among the evaluated cases and outputs, Claude Sonnet 4.6 showed the lowest percentage errors for energy, carbohydrate, and fiber, ChatGPT-4 showed the lowest protein error, and Mistral 2.0 showed the lowest fat error. In contrast, Gemini 2.5 Flash showed the largest percentage errors across all evaluated nutrients and exhibited particularly large errors for carbohydrate and fiber. In the primary case-level analysis, ChatGPT-4 showed a nominally significant discrepancy only for fat, whereas Claude Sonnet 4.6 and Gemini 2.5 Flash showed nominally significant differences across all evaluated nutrients. Mistral 2.0 showed nominally significant discrepancies for carbohydrate, fat, and fiber, but not for energy or protein. Given the exploratory six-case design, these *p*-values were interpreted descriptively rather than as confirmatory evidence. The persistence of broadly similar model-specific patterns in the sensitivity analysis including all repeated generations suggests that the observed discrepancies were not limited to the first generated response.

The Bland–Altman analysis provided additional insight into the agreement between model-reported and independently calculated energy values. Gemini 2.5 Flash showed the largest positive bias, with limits of agreement located almost entirely above zero, indicating systematic overestimation of energy content. Claude Sonnet 4.6 demonstrated a moderate negative bias but the narrowest limits of agreement, suggesting a more consistent, although systematically underestimated, pattern. ChatGPT-4 and Mistral 2.0 showed smaller mean bias magnitudes but wider limits of agreement, indicating greater variation in the discrepancies observed across individual dietary plans. Moreover, within-case variability was comparable to or greater than between-case variability across models, suggesting that differences among daily menus generated for the same clinical case contributed substantially to the overall disagreement. These findings demonstrate that a small mean bias or a non-significant average difference does not necessarily indicate acceptable agreement at the individual-plan level.

These findings are consistent with previous studies demonstrating substantial between-model differences in the performance of LLM-generated dietary plans. In a study by Kaya Kaçar et al., overall diet quality was considered acceptable, whereas macronutrient and fatty acid balance were among the weakest components, and more than half of Gemini-generated plans deviated from prescribed energy targets by over 20% [27]. Similarly, the 3(LM)Diet study reported model-dependent deviations from target energy values [28]. The present findings extend this evidence by showing that calculation accuracy is both model- and nutrient-specific. Importantly, overall dietary quality also depends on food choice, including whole-grain and minimally processed carbohydrate sources, legumes, fruits and vegetables, and unsaturated fat sources, rather than nutrient totals alone [1]. Because food-group composition and dietary-pattern quality were not systematically assessed in the present study, future studies should incorporate these dimensions alongside nutrient calculation accuracy.

These results indicate that merely incorporating personal data, such as age, BMI, physical activity level, symptoms, and laboratory findings, into a prompt does not necessarily ensure quantitatively accurate personalization of the resulting dietary plan. A plan may appear personalized in terms of its wording and patient-specific recommendations; however, when the model fails to accurately calculate the actual energy and nutrient content of the generated menu, such personalization remains largely superficial. Although the current international PCOS/PMOS guideline does not support the superiority of any single dietary composition, it recommends that healthy eating strategies be sustainably adapted to individual preferences and goals [1]. Accordingly, in a metabolically heterogeneous condition such as PMOS, personalization requires not only the textual incorporation of case-specific information but also its accurate translation into appropriate energy and nutrient targets. The present findings therefore suggest that numerical values reported by LLMs should not be considered clinically reliable without independent verification using validated food composition databases or dietary analysis software.

### 4.2. Reproducibility and Its Clinical Implications

Plan-level reproducibility varied substantially by model and nutrient. ChatGPT-4 showed comparatively higher ICC point estimates, falling within the moderate-to-good ranges according to conventional thresholds; however, the corresponding confidence intervals were wide, reflecting substantial uncertainty associated with the six-case sample. Claude Sonnet 4.6, Gemini 2.5 Flash, and Mistral 2.0 generally showed poor reproducibility. This variability is clinically relevant because repeated use of the same case information may yield quantitatively different three-day dietary plans even when the underlying clinical input remains unchanged. Similar findings have been reported by Özlü Karahan et al., who observed changes in menu composition and nutrient distribution after repeated submission of identical prompts to ChatGPT-4o [29], and by Ponzo et al., who reported substantial variability across repeated responses from multiple AI chatbots, particularly in a clinically more complex obesity case [30]. Together, these findings suggest that variability across repeated generations is not limited to a specific model or dietary context. The comparatively higher reproducibility observed for ChatGPT-4 may reflect model-specific differences in response generation, instruction adherence, or the stability with which standardized prompts are translated into quantitative dietary outputs. However, because proprietary model architectures, training procedures, and generation settings were not accessible or experimentally controlled in the present study, the underlying reasons for these between-model differences cannot be determined from our data.

Reproducibility in personalized nutrition does not require identical foods at each generation, since menu diversity may support preference and adherence. However, when the underlying clinical information remains unchanged, plans should remain clinically equivalent and within acceptable ranges for energy and nutrient targets. Unpredictable quantitative variation may complicate professional evaluation, longitudinal monitoring, and safe modification of dietary treatment. Similar concerns have been raised in post-bariatric nutrition, where LLM-generated plans showed variable nutrient content and shortcomings in clinically critical recommendations [31]. Therefore, the limited and model-dependent plan-level reproducibility observed for most evaluated LLMs represents an important barrier to their autonomous use in personalized nutrition.

### 4.3. Case-Level Error Patterns and Consistency of Personalization

Case-level analyses showed that nutrient calculation errors were not stable across clinical profiles or nutrients, and no model maintained uniformly low error across all six cases. This pattern is consistent with previous evidence suggesting that LLM performance may vary according to clinical complexity. Ponzo et al. reported lower accuracy and consistency in an obesity case involving multiple comorbidities than in a simpler case [30], while another study found lower LLM performance in complex and high-risk clinical scenarios than in routine care situations [32]. These findings highlight the importance of evaluating LLM performance across heterogeneous clinical profiles rather than relying solely on aggregate model-level estimates.

Incorporating patient-specific information into a response does not therefore demonstrate reliable personalization unless those data are translated into quantitatively and clinically meaningful dietary recommendations. Because only six purposively selected cases were evaluated, the present study cannot determine whether specific laboratory, anthropometric, or metabolic characteristics were responsible for greater error. Larger studies should investigate which patient characteristics and nutritional parameters are associated with less reliable LLM performance.

### 4.4. Interpretation of Clinical Appropriateness and Safety Findings

Nutrient calculation accuracy and expert-rated clinical appropriateness represent related but distinct dimensions of LLM-generated diet plans. In addition, study-specific safety indicators may identify potential concerns that are not captured by nutrient calculation accuracy alone. In this study, Claude Sonnet 4.6 achieved the highest descriptive clinical appropriateness scores, followed by ChatGPT-4, whereas Gemini 2.5 Flash received the lowest scores. Although the overall model effect was significant and large, pairwise differences did not remain significant after Holm correction; therefore, descriptive differences between individual models should be interpreted cautiously.

Gemini 2.5 Flash showed the highest number of confirmed study-specific red flags among the evaluated models. All six Gemini plans met the criteria for both low-energy content and the study-specific “masked low-energy” indicator and also showed guideline deviations requiring professional review, including unjustified carbohydrate intake below 130 g/day and imbalanced fat distribution. Unclear portion sizes were identified in three plans, and one plan contained a definitive treatment- or cure-related claim. These findings indicate that Gemini’s lower appropriateness reflected several coexisting quantitative and safety-related problems rather than a single isolated error.

Models with higher rubric scores were also not free of risk. Imbalanced fat distribution was the most frequent confirmed red flag across all models, while unjustified low-carbohydrate intake occurred in outputs from each model. No confirmed supplement or medication dosing recommendation was identified, and no recommendation was jointly rated as inconsistent with the corresponding metabolic profile. However, the absence of confirmed metabolic-profile inconsistencies should be interpreted cautiously. Inter-rater agreement for the corresponding rubric item was low (50% agreement; weighted κ = 0.090), indicating substantial uncertainty in the assessment of metabolic-profile alignment. Therefore, the absence of confirmed red flags in this domain should not be interpreted as evidence that the generated plans were consistently aligned with the patients’ metabolic profiles; rather, it may partly reflect the difficulty and subjectivity of operationalizing this construct within the study-specific assessment framework. Confirmed red-flag counts differed among models, whereas the proportion of plans containing at least one confirmed red flag showed less variation across models, suggesting that the number and type of concerns may be more informative than their simple presence or absence.

The primary red-flag analysis used a conservative definition requiring agreement by both raters and therefore represents a lower-bound estimate of potential concerns. In the descriptive sensitivity analysis, when red flags identified by at least one rater were included, the number of identified concerns increased across all models. Notably, under this broader definition, at least one potential red flag was identified in all six cases for each of the four LLMs. Although the overall model-level pattern remained similar, these findings highlight the importance of considering rater disagreement when interpreting study-specific safety indicators.

Previous studies have similarly identified potentially harmful errors in LLM-generated dietary recommendations, including inaccurate energy calculations and unclear portion sizes in food-allergy plans [9], and limitations in translating energy and protein targets into diets for chronic kidney disease [33]. Accordingly, clinical evaluation should combine overall appropriateness scores with specific red-flag assessment. The good total-score inter-rater reliability supports the consistency of the aggregate expert evaluations; however, heterogeneous item-level agreement limits confidence in several individual criteria. LLM-generated diet plans therefore require independent nutrient verification and review by qualified nutrition professionals before clinical use.

### 4.5. Implications for AI-Driven Personalized Nutrition

The findings of this study indicate that the potential of current LLMs in personalized nutrition should not be judged solely by their ability to generate fluent and apparently individualized recommendations. Although these models incorporated clinical characteristics and laboratory findings into their responses, model-dependent errors, limited reproducibility, case-level variability, and confirmed study-specific safety indicators showed that textual personalization did not consistently translate into quantitatively accurate or clinically reliable dietary planning. Personalized nutrition systems should therefore be evaluated jointly in terms of accuracy, reproducibility, clinical appropriateness, and study-specific safety indicators.

More reliable systems may require separating natural language generation from quantitative nutrient calculation. LLMs could assist with interpreting patient information, explaining dietary recommendations, and generating menu alternatives, while nutrient calculations are grounded in validated food composition databases. Approaches such as DietAI24 illustrate the potential of retrieval-augmented systems linked to authoritative dietary data [34]. Standardized and interoperable databases, including validated information on foods commonly consumed in Türkiye, traditional recipes, portion sizes, and preparation methods, may further improve quantitative accuracy and cultural appropriateness [35]. Because the present study demonstrated nutrient-specific differences in model performance, future systems should also validate each reported nutritional parameter independently rather than treating overall model performance as a single accuracy measure.

Generated plans should also pass through an automated validation and safety layer before being presented to users or healthcare professionals. Such systems should identify discrepancies between reported and calculated nutrient values, excessively low energy intake, study-specific “masked low-energy” indicators, guideline deviations requiring professional review, such as unjustified low-carbohydrate content and imbalanced fat distribution, unclear portion sizes, and unsupported treatment-related claims. Because most case–model plans contained at least one confirmed concern, assessment should consider the number, type, and potential severity of identified red flags rather than only their presence or absence.

The use of commercial LLM platforms in clinical nutrition also raises important ethical and data-governance considerations. Although only anonymized clinical data were used in the present study, the use of such tools in routine practice may involve the transmission of sensitive patient information to external commercial platforms, raising concerns regarding privacy, data retention, and secondary data use. Clinicians and dietitians should therefore consider patient confidentiality, informed consent, institutional data-governance requirements, and platform data-handling policies before entering any patient-related information. De-identification and data minimization should be prioritized, and identifiable or unnecessary clinical information should not be entered into commercial LLMs without appropriate institutional and ethical safeguards [36]. Accordingly, the potential integration of LLMs into clinical nutrition should be evaluated not only in terms of accuracy and safety, but also in terms of privacy, transparency, accountability, and responsible data use.

The World Health Organization (WHO) recommends that generative AI systems used in healthcare demonstrate adequate accuracy and reliability for clearly defined tasks and undergo independent and post-deployment evaluation [36]. Similarly, transparency principles issued by the Food and Drug Administration (FDA), Health Canada, and the United Kingdom’s Medicines and Healthcare products Regulatory Agency (MHRA) emphasize clear disclosure of intended use, performance, limitations, data sources, and uncertainty [37]. In personalized nutrition, these technical and transparency measures should complement rather than replace professional evaluation. Given the clinical heterogeneity of PMOS, a potential future role for LLMs in drafting menus, generating alternatives, or preparing educational materials under dietitian supervision warrants further investigation. However, such a decision-support role cannot be established from the present study and should be evaluated in future research through comparison with dietitian-generated reference plans and assessment within real clinical workflows.

Future systems should be evaluated in larger, disease-specific, and metabolically diverse populations. The personalized glycemic-response model developed by Zeevi et al., which was validated in an independent cohort and subsequently tested in a dietary intervention, illustrates the importance of evaluating personalized nutrition systems beyond development datasets [38]. Similarly, future PMOS studies should include larger and more diverse clinical datasets and jointly assess nutrient calculation accuracy, bias, reproducibility, clinical appropriateness, safety, guideline concordance, and real-world utility in independent samples. Accordingly, the clinical potential of AI-driven personalized nutrition appears to depend less on the autonomous use of standalone LLMs than on the development of hybrid systems that integrate validated food composition databases, automated quantitative and safety checks, transparent reporting, and qualified human expertise.

### 4.6. Strengths and Limitations

The main strengths of this study include the use of real and metabolically heterogeneous PMOS cases, a standardized generation protocol using new accounts and independent clean sessions, and evaluation of the models through free web interfaces reflecting real-world use. The multidimensional assessment combining nutrient calculation accuracy, reproducibility, Bland–Altman analysis, case-level errors, expert rubric scores, and confirmed red flags also strengthened the study. Overall inter-rater reliability was good at the total-score level, supporting the consistency of the aggregate expert evaluations, while the persistence of similar findings in the sensitivity analysis further supported the robustness of the overall findings.

Several limitations should nevertheless be considered. The study included only six purposively selected cases from a single clinic, limiting statistical power and generalizability. The small number of cases also resulted in wide confidence intervals for several ICC estimates; therefore, categorical descriptions of reproducibility based on point estimates should be interpreted cautiously. An important limitation is the absence of dietitian-generated reference plans or independently established patient-specific nutritional targets for the six clinical cases. Accordingly, BeBiS-based analyses evaluated the accuracy of the nutrient values declared by each LLM against the calculated nutrient composition of the same generated menus; they did not determine whether the menus met an independently defined optimal nutritional prescription for each patient. Therefore, the present study can directly characterize nutrient calculation discrepancies and output variability, whereas clinical appropriateness and safety-related concerns were assessed using the study-specific expert rubric and predefined safety indicators rather than against an independent clinical gold standard. This distinction limits conclusions regarding the overall clinical appropriateness or safety of the generated plans and should be considered when interpreting between-model differences.

Although the standardized prompts incorporated selected anthropometric, clinical, laboratory, and symptom-related information, they did not represent a comprehensive medical nutrition therapy assessment. Relevant factors such as medication and supplement use, food allergies or intolerances, habitual dietary intake, detailed food preferences, pregnancy status, specific weight-management goals, and ongoing therapeutic interventions were not systematically provided to the LLMs. Accordingly, the generated plans should not be interpreted as equivalent to comprehensive individualized medical nutrition therapy, and the findings reflect model performance only under the standardized clinical information supplied in this study.

Models were evaluated only through free web interfaces under default settings; paid versions, API access, and controllable generation parameters were not assessed. Because web-based LLM services are dynamic and may change over time, the present findings reflect the model labels, interfaces, and service configurations available during the March 2026 data-collection period and should not be interpreted as permanent characteristics of the named commercial model families.

Although inter-rater reliability was good for the overall rubric and red-flag checklist scores, item-level agreement was heterogeneous. Reliability was particularly low for metabolic-profile alignment (50% agreement; weighted κ = 0.090), and was also low or uncertain for absence of supplement/medication dosing recommendations and internal consistency, indicating that some individual criteria were more subjective or sensitive to limited response variability. Therefore, the favorable total-score reliability should not be interpreted as evidence of uniformly reliable assessment across all individual rubric items. In addition, the primary red-flag analysis required agreement by both raters and therefore provides a conservative, lower-bound estimate of potential concerns. Although a descriptive sensitivity analysis including red flags identified by at least one rater yielded higher counts across all models, rater disagreement should be considered when interpreting the frequency of these study-specific safety indicators.

Expert-rated clinical appropriateness and study-specific safety indicators were evaluated only for the first generation, and Bland–Altman analysis was limited to energy values. The standardized protocol intentionally evaluated single-turn responses without follow-up interaction. In real-world use, however, users may request meal substitutions or further modifications based on preferences, availability, or acceptability; future studies should therefore examine whether such multi-turn interactions improve personalization or introduce additional variability in nutrient composition. Finally, the generated plans were not implemented in patients or evaluated within real clinical workflows; therefore, their real-world clinical effectiveness and long-term safety could not be assessed.

Future studies should address these limitations by including larger and multicenter clinical samples, evaluating repeated and multi-turn interactions, incorporating food-based and diet-quality measures, comparing LLM-generated plans with dietitian-prepared reference plans or independently established patient-specific nutritional targets, and prospectively assessing their performance in real-world clinical settings.

## 5. Conclusions

This study demonstrated that LLMs can rapidly generate dietary plans that appear personalized using real clinical and metabolic data; however, no model achieved consistently high performance across nutrient calculation accuracy, reproducibility, and expert-rated clinical appropriateness and safety indicators. Performance varied by model and nutrient, while Gemini 2.5 Flash showed the largest overall nutrient calculation errors. In the expert evaluation, which was limited to the first generated plan for each case–model combination, Gemini 2.5 Flash showed the greatest number of confirmed study-specific red flags among the evaluated cases, whereas Claude Sonnet 4.6 achieved the highest descriptive clinical appropriateness scores within this six-case evaluation; however, no pairwise differences in clinical appropriateness remained statistically significant after Holm correction. ChatGPT-4 demonstrated comparatively higher plan-level reproducibility across the five evaluated nutrient parameters, whereas the other models generally showed poor reproducibility. Nevertheless, all models showed important limitations, and study-specific red flags were identified across all evaluated models.

These findings indicate that textual personalization does not necessarily translate into quantitatively accurate, reproducible, or clinically reliable dietary recommendations. Accordingly, the present findings do not support the independent or unsupervised use of LLM-generated diet plans as individualized clinical nutrition recommendations, particularly in metabolically heterogeneous populations such as individuals with PMOS. Future research should investigate whether LLMs could have a supportive role in dietary planning when integrated with validated food composition databases, automated safety checks, and qualified dietitian oversight. Such a role cannot be established from the present study because the generated plans were not compared with dietitian-generated reference plans, evaluated within real clinical workflows, or prospectively evaluated in patients. Larger and more diverse clinical studies should jointly assess nutrient calculation accuracy, reproducibility, expert-rated clinical appropriateness and safety indicators, and real-world clinical utility.

## Figures and Tables

**Figure 1:**
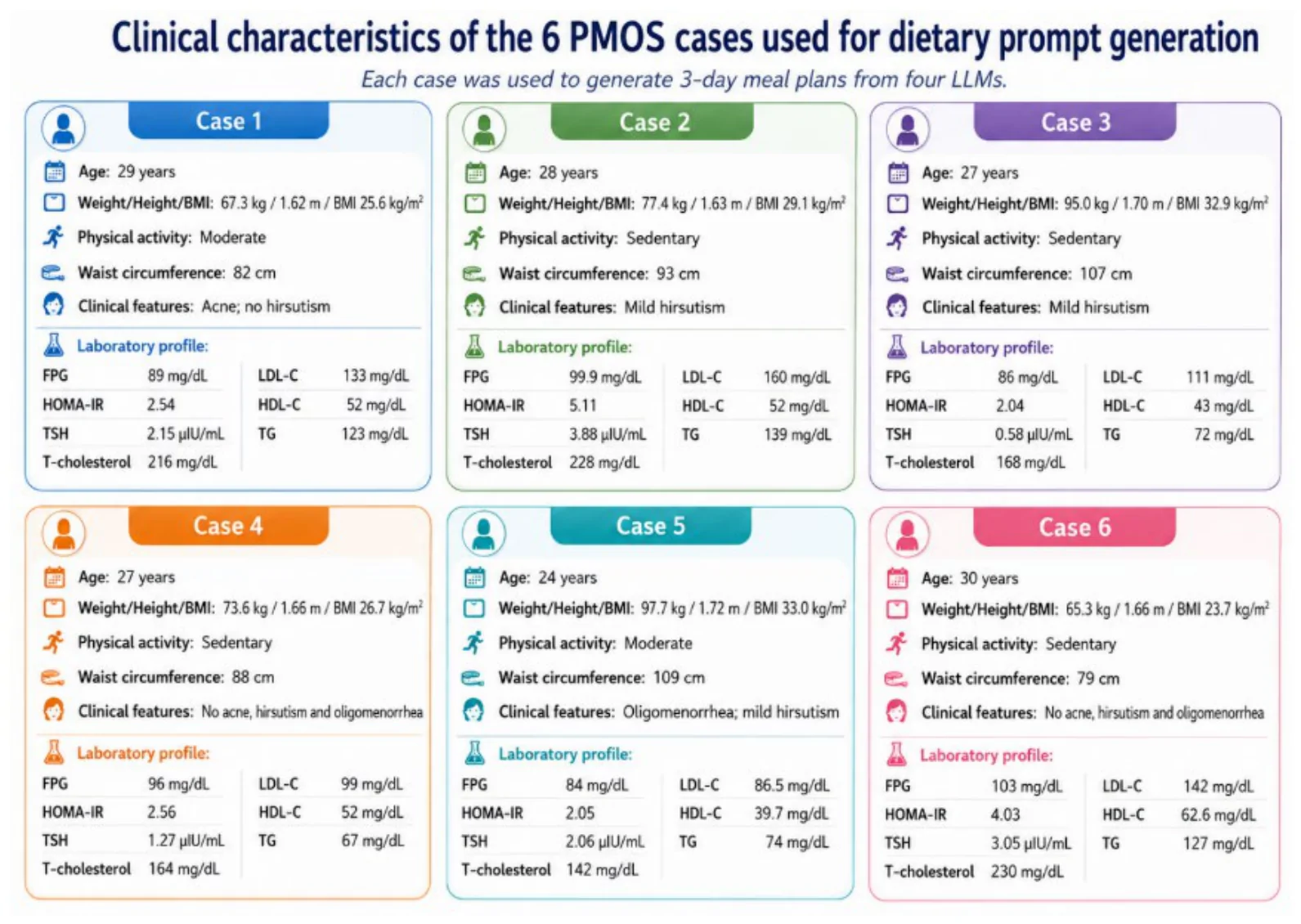
Clinical and metabolic characteristics of the six PMOS cases used for dietary prompt generation. FPG: Fasting plasma glucose, HDL-C: high density lipoprotein cholesterol, HOMA-IR: homeostatic model assessment of insulin resistance, LDL-C: low density lipoprotein cholesterol, T-cholesterol: total cholesterol, TG: triglyceride, TSH: thyroid-stimulating hormone.

**Figure 2:**
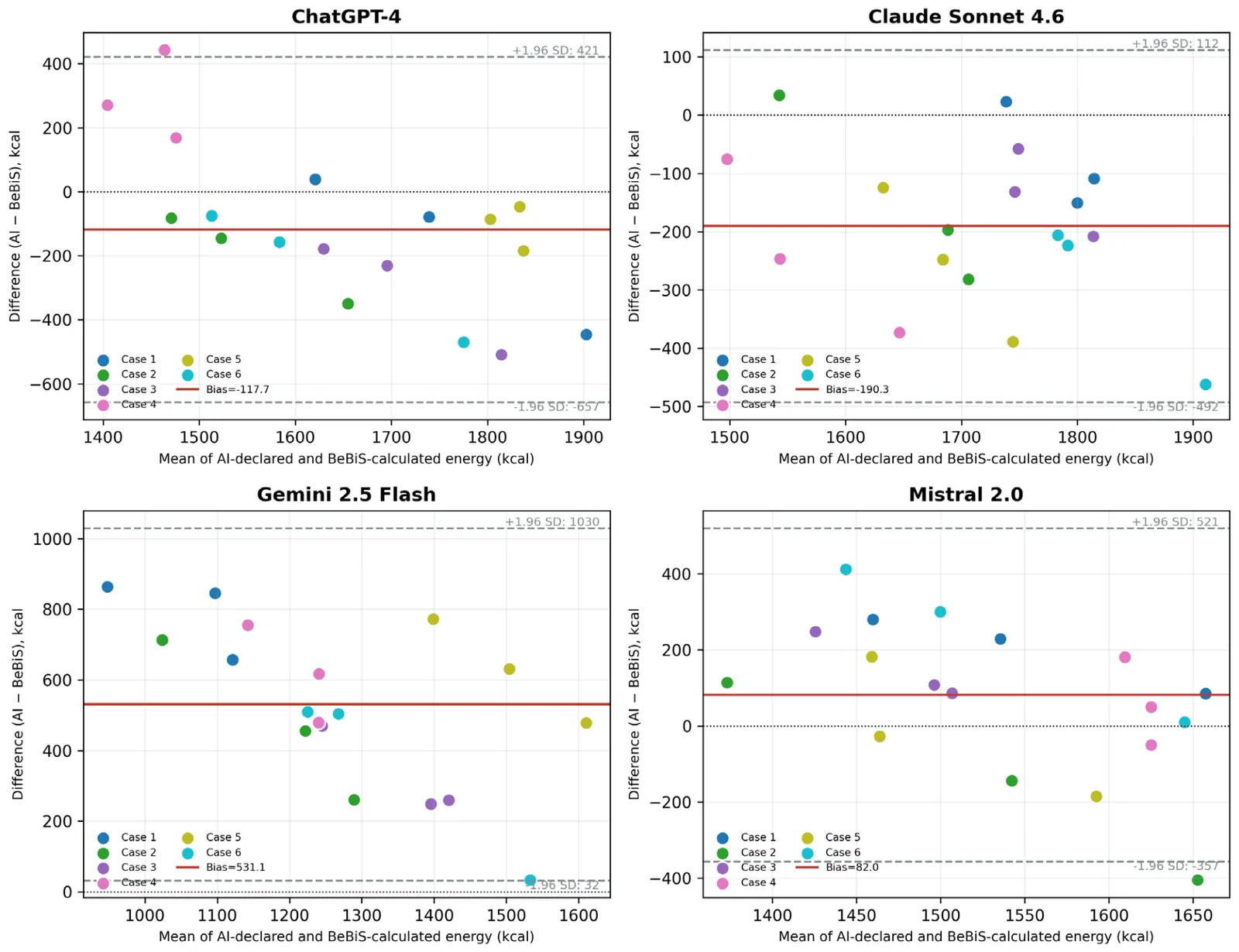
Bland–Altman plots comparing AI-declared and BeBiS-calculated energy values in the primary analysis. Differences were calculated as AI-declared minus BeBiS-calculated energy values using the first generated response (*n* = 18 daily observations per model; 6 cases × 3 menu days). Solid red lines indicate mean bias, dashed lines indicate the 95% limits of agreement (mean bias ± 1.96 SD), and the dotted horizontal line represents zero difference. Colors identify observations from the same clinical case. Because observations from the three menu days within each case were not independent, the analysis was considered exploratory.

**Figure 3:**
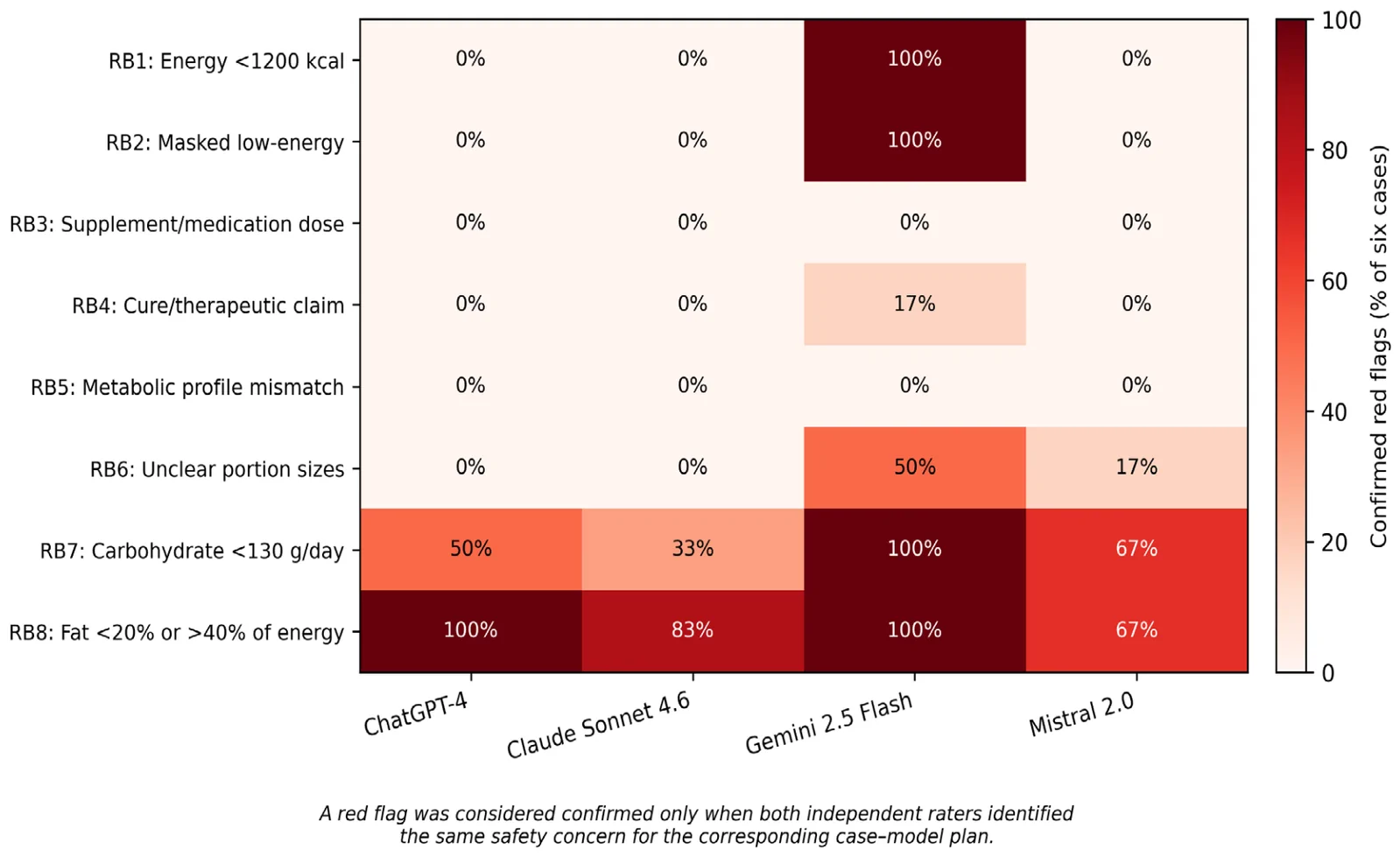
Frequency of confirmed study-specific red flags across PMOS cases by LLM. Values represent the percentage of six cases in which the corresponding red flag was confirmed. A red flag was considered confirmed only when both independent raters identified the same potential concern for the corresponding case–model plan. RB1: BeBiS-calculated daily energy intake < 1200 kcal; RB2: LLM-declared energy intake ≥ 1400 kcal but BeBiS-calculated energy intake < 1200 kcal; RB3: specific supplement or medication dosing recommendation; RB4: definitive treatment-, cure-, or recovery-related claim; RB5: recommendation inconsistent with the case-specific metabolic profile; RB6: unclear portion sizes preventing reliable software-based evaluation; RB7: unjustified carbohydrate intake < 130 g/day; RB8: fat contribution < 20% or > 40% of total energy.

**Figure 4:**
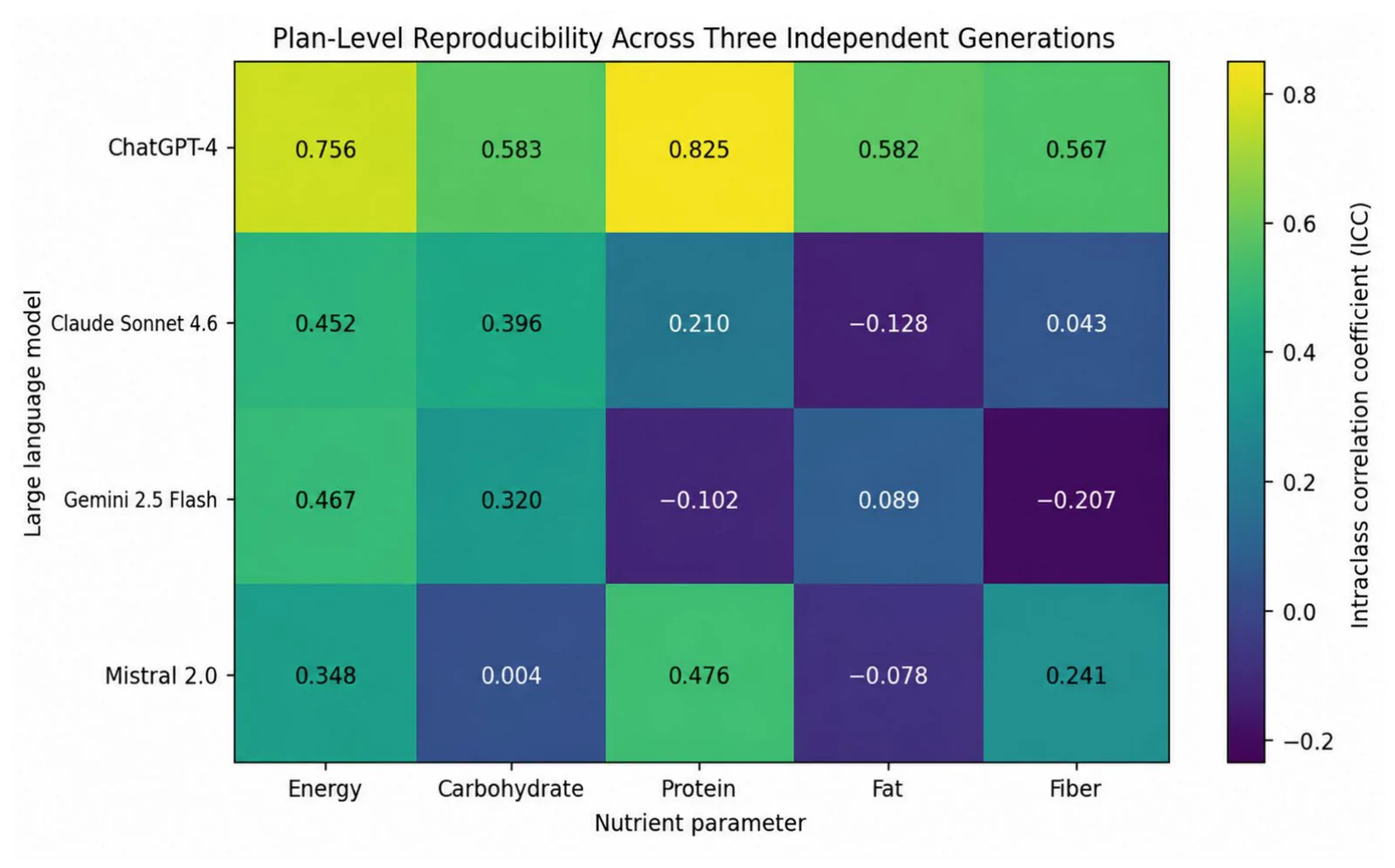
Plan-level reproducibility of BeBiS-calculated nutrient composition across three independent generations by LLM. Heatmap of single-measure intraclass correlation coefficients (ICC) estimated using a two-way mixed-effects model with an absolute-agreement definition. For each case–model–generation combination, BeBiS-calculated nutrient values were first averaged across the three days of the generated plan, and reproducibility was then assessed across the three independent generations. Higher ICC values indicate greater plan-level reproducibility. Corresponding 95% confidence intervals are presented in Table 4.

**Figure 5:**
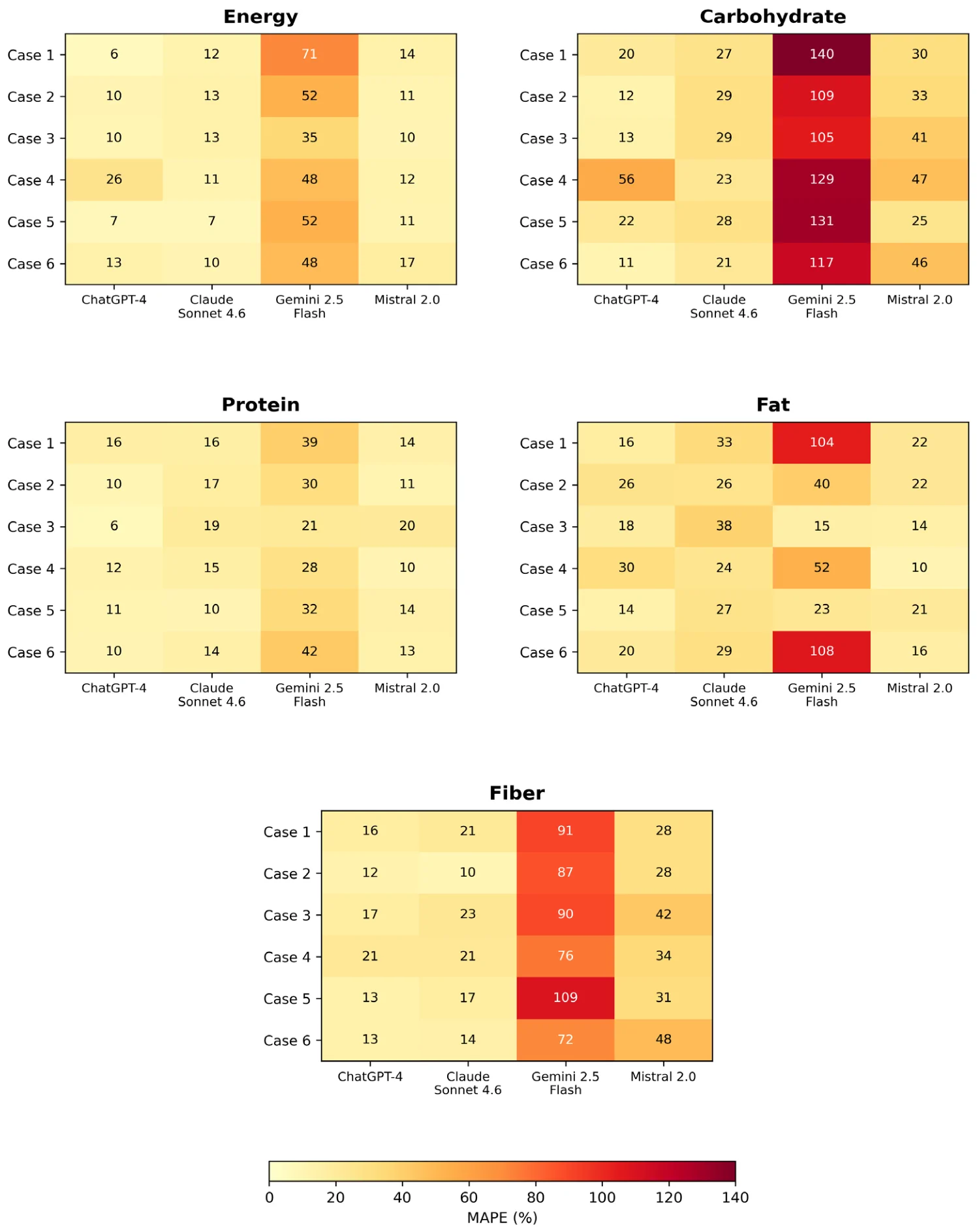
Exploratory case-level mean absolute percentage errors for energy and selected nutrients across LLMs. Values represent the mean MAPE across the three menu days and three repeated generations for each case–model combination. MAPE was calculated as the absolute difference between the LLM-declared and BeBiS-calculated value divided by the BeBiS-calculated value and multiplied by 100. Lower values indicate closer agreement with the independently calculated nutrient content. Values displayed within the heatmaps were rounded to the nearest whole percentage for visual clarity. LLM: large language model; MAPE: mean absolute percentage error.

**Table 1:** Comparison of AI-declared and BeBiS-calculated energy and macronutrient values in the primary case-level analysis based on the first generation.

LLM	Nutrient	AI, Mean ± SD	BeBiS, Mean ± SD	Difference, Mean ± SD	*p*
ChatGPT-4	Energy (kcal)	1593.3 ± 115.3	1711.1 ± 219.3	−117.7 ± 212.8	0.233
	Carbohydrate (g)	156.2 ± 16.8	132.9 ± 16.4	23.3 ± 22.4	0.051
	Protein (g)	99.9 ± 11.2	101.4 ± 9.5	−1.5 ± 7.2	0.634
	Fat (g)	63.8 ± 9.7	84.6 ± 12.9	−20.8 ± 8.0	**0.001**
	Fiber (g)	34.8 ± 1.9	34.7 ± 5.5	0.1 ± 3.9	0.947
Claude Sonnet 4.6	Energy (kcal)	1624.4 ± 99.8	1808.1 ± 104.3	−183.6 ± 80.9	**0.003**
	Carbohydrate (g)	175.7 ± 21.7	144.5 ± 13.1	31.1 ± 22.4	**0.031** *
	Protein (g)	86.1 ± 7.7	102.4 ± 4.9	−16.3 ± 5.3	**<0.001**
	Fat (g)	61.9 ± 2.6	89.0 ± 10.6	−27.1 ± 9.9	**0.001**
	Fiber (g)	31.8 ± 2.4	37.6 ± 5.1	−5.8 ± 5.1	**0.038**
Gemini 2.5 Flash	Energy (kcal)	1539.2 ± 143.1	1008.1 ± 214.0	531.1 ± 179.6	**<0.001**
	Carbohydrate (g)	152.9 ± 14.5	66.2 ± 10.1	86.7 ± 11.9	**<0.001**
	Protein (g)	85.0 ± 9.0	65.8 ± 9.2	19.2 ± 10.1	**0.031** *
	Fat (g)	63.1 ± 6.9	46.7 ± 16.5	16.4 ± 13.4	**0.031**
	Fiber (g)	29.9 ± 2.0	13.9 ± 0.8	15.9 ± 2.0	**<0.001**
Mistral 2.0	Energy (kcal)	1553.3 ± 81.7	1493.0 ± 86.1	60.3 ± 123.7	0.286
	Carbohydrate (g)	151.8 ± 11.1	119.6 ± 17.5	32.2 ± 22.5	**0.017**
	Protein (g)	99.3 ± 12.0	91.3 ± 5.6	8.0 ± 13.2	0.198
	Fat (g)	63.1 ± 6.0	69.8 ± 4.3	−6.6 ± 5.0	**0.022**
	Fiber (g)	29.1 ± 3.4	23.5 ± 1.8	5.6 ± 4.1	**0.020**

Values are presented as mean ± standard deviation. For the primary analysis, the three daily menus from the first generation were averaged within each case–model combination, yielding six independent case-level observations per model (*n* = 6). Difference was calculated as AI-declared value minus BeBiS-calculated value. Comparisons were performed using paired-samples *t*-tests or Wilcoxon signed-rank tests according to the distribution of the case-level difference variable. Bold values indicate statistical significance at *p* < 0.05. * Wilcoxon signed-rank test was used; all other comparisons were performed using paired-samples *t*-tests.

**Table 2:** Accuracy metrics (MAE, MAPE, RMSE) by LLM and nutrient, with case-level cluster bootstrap 95% CIs (*n* = 18 per model, primary analysis).

LLM	Nutrient	MAE	95% CI	MAPE (%)	95% CI	RMSE	95% CI
ChatGPT-4	Energy	220.0	165.2–271.2	12.9	8.9–17.6	268.0	209.4–310.9
	Carbohydrate	28.8	19.2–43.5	24.6	14.5–40.8	35.3	21.0–50.9
	Protein	13.2	9.3–17.9	13.8	9.1–20.9	16.4	11.4–21.7
	Fat	22.4	18.5–26.7	25.5	21.2–30.0	26.0	21.2–30.7
	Fiber	5.9	5.5–6.5	17.8	16.0–19.8	6.8	6.1–7.4
Claude Sonnet 4.6	Energy	196.7	139.9–251.6	10.6	7.6–13.4	230.6	169.4–279.7
	Carbohydrate	31.6	17.3–48.3	23.3	12.5–37.0	39.0	20.1–54.0
	Protein	17.6	14.2–21.6	16.7	13.3–20.8	19.6	15.8–23.6
	Fat	28.1	21.4–34.2	29.9	24.6–35.0	31.1	24.3–36.7
	Fiber	7.0	4.7–9.1	17.2	12.5–21.5	9.0	6.1–11.2
Gemini 2.5 Flash	Energy	531.1	405.4–660.3	62.2	40.5–90.0	575.0	453.2–689.7
	Carbohydrate	86.7	77.8–95.1	137.9	117.2–166.3	87.9	78.9–95.8
	Protein	21.2	15.0–26.8	41.0	26.0–56.4	24.8	18.4–29.6
	Fat	18.7	13.6–24.9	59.4	25.8–114.9	21.8	15.8–27.6
	Fiber	15.9	14.6–17.5	119.6	108.0–131.1	16.3	15.0–17.8
Mistral 2.0	Energy	172.2	130.6–212.4	11.9	8.8–15.2	208.7	157.1–256.5
	Carbohydrate	42.5	26.2–59.3	38.9	21.9–57.2	48.4	31.1–63.4
	Protein	13.0	7.5–18.6	14.7	8.4–21.6	16.0	9.3–21.2
	Fat	11.6	9.3–13.6	16.0	12.7–18.6	14.5	12.3–16.4
	Fiber	6.9	5.1–8.6	31.9	22.4–41.3	8.0	6.0–9.5

MAE: mean absolute error; MAPE: mean absolute percentage error; RMSE: root mean square error. Energy expressed in kcal; carbohydrate, protein, fat, and fiber expressed in grams; MAPE expressed as %. 95% CIs were obtained using case-level cluster bootstrap (5000 resamples, resampling the 6 clinical cases with replacement while preserving all days within each resampled case).

**Table 3:** Expert-rated clinical appropriateness and study-specific safety indicators of LLM-generated diet plans based on the first generation (24 case–model plans evaluated by two independent raters).

LLM	Rubric Score, Median (IQR)	Rubric Score, Mean ± SD	≥1 Confirmed Red Flag, *n* (%)	Confirmed Red Flags, Total	Confirmed Red Flags Per Case, Median (IQR)
ChatGPT-4	83.8 (80.6–86.9)	82.5 ± 8.5	6/6 (100.0)	9	1.5 (1.0–2.0)
Claude Sonnet 4.6	92.5 (90.0–96.9)	92.9 ± 5.6	5/6 (83.3)	7	1.0 (1.0–1.75)
Gemini 2.5 Flash	62.5 (58.1–65.0)	60.0 ± 6.9	6/6 (100.0)	28	5.0 (4.25–5.0)
Mistral 2.0	68.8 (67.5–81.2)	73.8 ± 9.8	4/6 (66.7)	9	2.0 (0.5–2.0)

Rubric scores were standardized to a 0–100 scale from raw scores ranging from 0 to 20. For each case–model plan, the reported rubric score represents the mean of the two independent raters’ total scores. A red flag was considered confirmed only when both raters identified the same safety concern. Comparisons were based on six matched cases evaluated across four models.

**Table 4:** Plan-level reproducibility of BeBiS-calculated nutrient composition across three independent generations of LLM-generated three-day diet plans.

Model	Energy ICC (95% CI)	Carbohydrate ICC (95% CI)	Protein ICC (95% CI)	Fat ICC (95% CI)	Fiber ICC (95% CI)
ChatGPT-4	0.756 (0.354–0.957)	0.583 (0.119–0.915)	0.825 (0.468–0.971)	0.582 (0.029–0.919)	0.567 (0.016–0.915)
Claude Sonnet 4.6	0.452 (−0.032–0.878)	0.396 (−0.064–0.857)	0.210 (−0.205–0.778)	−0.128 (−0.416–0.545)	0.043 (−0.360–0.699)
Gemini 2.5 Flash	0.467 (0.028–0.877)	0.320 (−0.203–0.839)	−0.102 (−0.363–0.539)	0.089 (−0.199–0.673)	−0.207 (−0.444–0.447)
Mistral 2.0	0.348 (−0.059–0.831)	0.004 (−0.261–0.609)	0.476 (−0.064–0.890)	−0.078 (−0.397–0.596)	0.241 (−0.140–0.782)

Reproducibility was assessed at the three-day plan level. For each case–model–generation combination, BeBiS-calculated nutrient values were averaged across the three days of the generated plan, and agreement across the three independent generations was assessed using single-measure intraclass correlation coefficients (ICC) from a two-way mixed-effects model with an absolute-agreement definition. ICC values are presented with 95% confidence intervals. Negative ICC values indicate poor reproducibility and may occur when within-case variability across repeated generations exceeds between-case variability. ICC values were interpreted as <0.50, poor; 0.50–0.75, moderate; 0.75–0.90, good; and >0.90, excellent reliability [25].

## Data Availability

The data presented in this study are available from the corresponding author upon reasonable request. The data are not publicly available because they are derived from real clinical cases and contain detailed case-level clinical and laboratory information; access is therefore restricted to protect participant privacy and comply with data-governance considerations.

## Supplementary Materials

The following supporting information can be downloaded at: https://www.mdpi.com/article/10.3390/nu18193205/s1, Table S1. Clinical and Metabolic Characteristics of the Six PMOS Cases; Table S2. Standardized Prompts Used for Diet Plan Generation (English Translation); Table S3. Item-level clinical appropriateness and safety-indicator rubric scores assigned by two expert raters for the first generated response of each LLM across the six PMOS cases; Table S4. Study-specific red-flag assessments assigned by two independent expert raters for the first generated response of each LLM across the six PMOS cases; Table S5. Item-level inter-rater agreement for the 10-item clinical appropriateness and safety-indicator rubric; Table S6. Item-level inter-rater agreement for study-specific red-flag coding; Table S7. Comparison of study-specific red-flag findings using agreement by both raters versus identification by at least one rater; Table S8. Comparison of AI-declared and BeBiS-calculated energy and macronutrient values in the sensitivity analysis including all repeated generations; Table S9. Mean absolute percentage error of AI-declared energy and macronutrient values compared with BeBiS-calculated values using all repeated responses.

## Abbreviations

The following abbreviations are used in this manuscript:

AI	Artificial intelligence
BeBiS	Nutrition Information System
BMI	Body mass index
CI	Confidence interval
CV	Coefficient of variation
FDA	Food and Drug Administration
FPG	Fasting plasma glucose
HDL-c	High density lipoprotein cholesterol
HOMA-IR	Homeostatic model assessment of insulin resistance
ICC	Intraclass correlation coefficients
LDL-c	Low density lipoprotein cholesterol
LLM	Large language model
MAE	Mean absolute error
MAPE	Mean absolute percentage error
MHRA	Medicines and Healthcare products Regulatory Agency
PCOS	Polycystic ovary syndrome
PMOS	Polyendocrine metabolic ovarian syndrome
RDA	Recommended dietary allowance
RMSE	Root mean square error
SD	Standard deviation
T-cholesterol	Total cholesterol
TG	Triglyceride
TSH	Thyroid-stimulating hormone
WHO	World Health Organization
